# Profil épidémiologique de la tuberculose dans un contexte sécuritaire difficile (2021-2023): région du Centre-Est, Burkina Faso

**DOI:** 10.11604/pamj.2025.52.119.49410

**Published:** 2025-11-20

**Authors:** Saïdou-Mady Bagaya, Issiaka Bebane, Djibril Boro, Morou Nikiema, Ansiouonèkou Pascal Somda, Kouka Joseph Ouedraogo, Kobena Naon, Dahourou Sou, Issa Guire, Franck Palamanga Obulbiga, Denis Yelbeogo

**Affiliations:** 1 Direction Régionale de la Santé, Région du Centre-Est, Ministère de la Santé, Tenkodogo, Burkina Faso,; 2 Centre Hospitalier Régional de Tenkodogo, Région du Centre-Est, Ministère de la Santé, Tenkodogo, Burkina Faso,; 3 Centre des Opérations de Réponse aux Urgences Sanitaires, Ministère de la Santé, Ouagadougou, Burkina Faso,; 4 Coordination du Programme de Formation en Epidémiologie de Terrain, Frontline et Intermédiaire, Ouagadougou, Burkina Faso

**Keywords:** Tuberculose, épidémiologie, profession, accessibilité aux soins, Burkina Faso, Tuberculosis, epidemiology, occupation, health services accessibility, Burkina Faso

## Abstract

**Introduction:**

la tuberculose constitue toujours un problème majeur de santé publique au Burkina Faso. Dans la région du Centre-Est, le contexte sécuritaire dégradé entrave l'accès aux soins et complique les efforts de lutte. Cette étude vise à décrire le profil épidémiologique des cas enregistrés entre 2021 et 2023.

**Méthodes:**

il s'agit d'une étude transversale descriptive à partir des données de neuf centres de diagnostic et de traitement (CDT). L'échantillonnage était exhaustif et incluait tous les nouveaux cas et rechutes enregistrés du 1^er^ janvier 2021 au 31 décembre 2023. Les variables analysées portaient sur l'âge, le sexe, la profession, les caractéristiques cliniques et les résultats thérapeutiques.

**Résultats:**

un total de 1581 cas de tuberculose a été enregistré dont 92,6% étaient de nouveaux cas. L'âge médian des patients était de 31 ans (IQR: 22-44; étendue: 1 à 90 ans). La tranche d'âge de 25 à 34 ans représentait 27,8% des cas. Le sexe-ratio était de 3,8 en faveur des hommes (79,2%). Les orpailleurs (31,8%) et les agriculteurs (27,6%) étaient les plus représentés. La tuberculose pulmonaire représentait 87,9% des cas, dont 66,9% étaient confirmés bactériologiquement. Le taux global de succès thérapeutique était de 78,3%, et la létalité atteignait 9,1%.

**Conclusion:**

la tuberculose dans la région du Centre-Est touche surtout les jeunes adultes actifs, particulièrement les orpailleurs et les agriculteurs. Dans un contexte sécuritaire fragile, des stratégies mobiles, communautaires et résilientes sont nécessaires pour améliorer la détection et la prise en charge.

## Abstract (en)

**Introduction:**

tuberculosis remains a major public health problem in Burkina Faso. In the Centre-Est region, the deteriorating security situation hinders access to care and complicates control efforts. This study aims to describe the epidemiological profile of cases recorded between 2021 and 2023 in this region.

**Methods:**

a descriptive cross-sectional study was conducted using data from nine tuberculosis diagnosis and treatment centers (CDTs). The sampling was exhaustive and included all new cases and relapses recorded from January 1^st^, 2021, to December 31^st^, 2023. The variables analysed included age, sex, occupation, clinical characteristics, and treatment outcomes.

**Results:**

a total of 1,581 cases of tuberculosis were recorded, 92.6% of which were new cases. The median age of patients was 31 years (IQR: 22-44; range: 1 to 90 years). The 25-34 age group accounted for 27.8% of cases. The sex ratio was 3.8 in favor of men (79.2%). Gold miners (31.8%) and farmers (27.6%) were the most represented occupational groups. Pulmonary tuberculosis accounted for 87.9% of cases, of which 66.9% were bacteriologically confirmed. The overall treatment success rate was 78.3%, and the case fatality rate was 9.1%.

**Conclusion:**

tuberculosis in the Central-East region mainly affects young working adults, particularly gold miners and farmers. In a fragile security context, mobile strategies

## Introduction

La tuberculose est une infection à transmission aérienne causée par les bactéries du complexe *Mycobacterium tuberculosis*, responsables en grande partie des formes pulmonaires contagieuses [1]. Elle sévit dans toutes les régions du globe et demeure une cause majeure de morbidité et de mortalité. En 2023, on estime que 10,8 millions de personnes ont été touchées dans le monde, avec près de 1,25 million de décès [2]. Au Burkina Faso, la tuberculose persiste comme un problème prioritaire de santé publique, en dépit des efforts de prévention et de prise en charge. En 2024, l'incidence était estimée à 39,5 cas pour 100000 habitants et la létalité à 7,4% [3]. Au niveau national, la coordination des activités de lutte contre la tuberculose est assurée par le Programme national de lutte contre la tuberculose (PNT), mis en place en 1995. Au niveau régional, la Direction régionale de la santé (DRS), en partenariat avec les districts sanitaires, met en œuvre les interventions via les formations sanitaires de premier niveau [4]. Toutefois, dans la région du Centre-Est, la détérioration du climat sécuritaire depuis 2018 a entraîné la fermeture de plusieurs structures de santé, réduisant considérablement l'accès aux soins [5]. Cette situation entrave les actions de lutte et se traduit par des niveaux de morbidité et de mortalité encore préoccupants; en témoigne l'incidence régionale qui était estimée à 34 pour 100000 habitants, avec une létalité de 10,2% [3] en 2024.

Dans la littérature, plusieurs études décrivent le profil épidémiologique de la tuberculose, mais pour la plupart dans un contexte de paix. Afin de combler ce gap, il nous a paru nécessaire de diligenter la présente étude décrivant le profil épidémiologique de la tuberculose dans un contexte à défis sécuritaires. En outre, l'étude permettra de fournir des informations utiles à la planification et à l'adaptation des interventions de lutte dans un système de santé fragilisé par la crise sécuritaire.

## Méthodes

**Cadre de l'étude:** la présente étude a été menée dans la région du Centre-Est du Burkina Faso, qui couvre une superficie de 15288 km^2^ et comptait environ 1750461 habitants en 2023 [6]. La région est frontalière avec le Togo et le Ghana. Elle est divisée en trois provinces et compte un total de 30 communes, dont 24 rurales et six urbaines [7]. L'agriculture constitue à la fois l'activité principale et la base économique des ménages de la région [8]. L'orpaillage artisanal est également très présent et mobilise des milliers de travailleurs répartis sur plusieurs sites [9]. Sur le plan sanitaire, la région dispose d'un centre hospitalier régional (CHR), de six centres médicaux avec antennes chirurgicales (CMA), et de 215 centres de santé et de promotion sociale (CSPS), répartis dans sept districts sanitaires que sont: Ouargaye, Bittou, Zabré, Koupèla, Pouytenga, Garango et Tenkodogo [3].

La lutte contre la tuberculose repose sur neuf CDT qui sont chargés du dépistage, de la confirmation diagnostique, de l'initiation et du suivi des traitements [4]. Les CSPS assurent la collecte des échantillons, le suivi communautaire des patients et la transmission des données aux CDT [4]. Toutefois, l'insécurité persistante dans certaines zones, notamment à Ouargaye et Bittou, limite l'accès aux services et perturbe la continuité des activités de dépistage et de prise en charge [5]. En plus de la fermeture des formations sanitaires, la situation sécuritaire a occasionné des déplacements de population vers les zones stables. Ce qui entraine une promiscuité, propice à la transmission du bacille de Koch.

**Type et période d'étude:** il s'agit d'une étude transversale descriptive réalisée sur trois ans, du 1^er^ janvier 2021 au 31 décembre 2023.

**Population d'étude:** l'étude a inclus tous les patients pris en charge pour tuberculose dans les formations sanitaires de la région du Centre-Est entre 2021 et 2023.

**Échantillonnage:** un échantillonnage exhaustif a été effectué, incluant l'ensemble des cas enregistrés dans neuf (9) CDT sur la période d'étude: deux CDT dans les districts de Garango et de Tenkodogo et un CDT dans chacun des six autres districts (Ouargaye, Bittou, Zabré, Pouytenga et Koupèla).

**Critères d'inclusion:** ont été inclus tous les patients enregistrés dans les CDT durant la période d'étude, diagnostiqués soit par confirmation bactériologique (examen microscopique, test Xpert MTB/RIF ou culture), soit par diagnostic clinique établi par un médecin.

**Critères de non-inclusion:** les cas de tuberculose multirésistante (résistance à au moins l'isoniazide et la rifampicine) n'ont pas été inclus.

**Sources des données:** les registres de tuberculose disponibles dans chacun des CDT de la région nous ont servi de source de collecte de données.

**Techniques et outils de collecte des données:** une analyse documentaire des registres de prise en charge de la tuberculose a été réalisée. Les données ont été saisies à l'aide d'un formulaire électronique conçu sur la plateforme KoboToolbox.

**Traitement et analyse des données:** l'analyse des données a été réalisée à l'aide du logiciel STATA, version 15.1. Une analyse descriptive a été effectuée sur les principales variables étudiées: caractéristiques sociodémographiques (âge, sexe, profession, lieu de résidence), caractéristiques cliniques (type de tuberculose, mode de confirmation) et résultats thérapeutiques. Les résultats sont présentés sous forme de proportions, de ratio homme/femme, de distribution par classes d'âge et d'évolution mensuelle et annuelle des cas.

**Considérations éthiques:** l'autorisation d'utiliser les données a été obtenue auprès du directeur régional de la santé du Centre-Est. Les données issues des registres ont été analysées de manière anonyme et confidentielle, sans possibilité d'identification individuelle. Cette étude s'inscrit dans le cadre de la surveillance épidémiologique de routine et ne nécessitait donc pas de consentement individuel, conformément aux recommandations éthiques nationales.

## Résultats

Durant la période allant du 1^er^ janvier 2021 au 31 décembre 2023, un total de 1581 cas de tuberculose ont été enregistrés dans la région du Centre-Est, dont 92,6% étaient de nouveaux cas.

**Caractéristiques sociodémographiques des patients:** l'âge médian des patients était de 31 ans (IQR: 22-44), avec une étendue de 1 à 90 ans. La tranche d'âge de 25 à 34 ans représentait 27,77% (439/1581) des cas. Le sexe-ratio homme/femme était de 3,8 (Tableau 1). Concernant la profession, les orpailleurs représentaient 31,82% (503/1581) des cas, suivis des agriculteurs 27,64% (437/1581). Les districts sanitaires de Zabré et de Tenkodogo ont enregistré respectivement 28,21% (446/1581) et 20,87% (330/1581) des cas, tandis que les districts d'Ouargaye et Bittou avaient enregistré respectivement 4,24% (67/1581) et 3,86% (61/1581). (Tableau 1).

**Tableau 1:** répartition des cas de tuberculose selon les caractéristiques sociodémographiques, région du Centre-Est, (2021-2023)

Caractéristiques	Effectif (n=1581)	Pourcentage (%)
**Tranche d'âge**	-	-
<15 ans	118	7,46
15-24 ans	351	22,20
25-34 ans	439	27,77
35-44 ans	260	16,45
≥45 ans	413	26,12
**Sexe**		
Homme	1252	79,19
Femme	329	20,81
**Profession**		
Agriculteur	437	27,64
Travailleurs salariés	37	2,34
Elève/Etudiant	28	1,77
Commerçant	214	13,54
Orpailleur	503	31,82
Sans emploi	362	22,90
**District sanitaire**	-	-
Bittou	61	3,86
Garango	165	10,44
Koupï¿½la	256	16,19
Ouargaye	67	4,24
Pouytenga	256	16,19
Tenkodogo	330	20,87
Zabré	446	28,21

**Figure 1:**
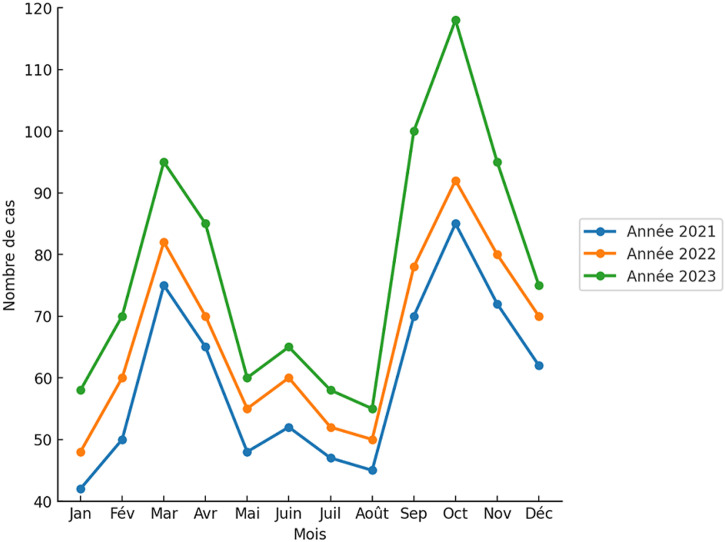
évolution mensuelle du nombre de cas de tuberculose selon l'issue thérapeutique de janvier à décembre, région du Centre-Est, (2021-2023)

**Évolution de l'incidence annuelle et mensuelle des cas:** l'analyse temporelle révèle une tendance à la hausse de l'incidence de la tuberculose entre 2021 et 2023, avec des taux passant de 29,31 pour 100000 habitants en 2021 à 29,82 en 2022, puis à 33,30 en 2023. Par ailleurs, deux pics saisonniers récurrents ont été observés chaque année, entre février et avril, puis entre septembre et novembre, suggérant une variabilité saisonnière possiblement liée aux fluctuations de la transmission ou à l'intensité du dépistage (Figure 1).

**Résultats du diagnostic et caractéristiques cliniques:** parmi les 1581 cas, 1058 (66,92%) ont été confirmés bactériologiquement, et 523 (33,08%) ont été diagnostiqués cliniquement. La forme pulmonaire représentait 1390 cas (87,92%), et 191 cas (12,08%) étaient des formes extra-pulmonaires (Tableau 2). Sur les 1581 cas de tuberculose enregistrés, le statut sérologique du virus de l'immunodéficience humaine (VIH) était connu pour 1564 patients. Parmi eux, 94 (6,01%) étaient séropositifs. Chez ces derniers, 83 cas (88,30%) avaient une tuberculose pulmonaire et 11 cas (11,70%) une forme extra-pulmonaire (Tableau 2)

**Tableau 2:** répartition des caractéristiques diagnostiques et cliniques selon le statut VIH, région du Centre-Est, (2021-2023)

Caractéristiques	VIH Positif n=94(%)	VIH négatif n=1470(%)
**Mode de diagnostic**	-	-
Confirmé bactériologiquement	62 (5,94)	982(94,06)
Diagnostiqué cliniquement	32 (6,15)	488 (93,85)
**Type de tuberculose**	-	-
Pulmonaire	83 (88,30)	1292 (87,89)
Extra-pulmonaire	11 (11,70)	178 (12,11)

**Résultats du traitement:** le taux global de succès thérapeutique était de 78,3% (1238/1581). Le taux d'échec s'élevait à 3,92% (62 patients/1581), le taux d'abandon à 8,67% (137 patients/1581) et le taux de décès atteignait 9,11% (144 patients/1581) (Tableau 3).

**Tableau 3:** répartition des patients tuberculeux par district sanitaire selon l'issue thérapeutique, région du Centre-Est, (2021-2023)

District sanitaire	Cas traités n=1581	Succès thérapeutique n(%)	Echec	Abandon (%)	Décès n(%)
Bittou	61	57 (93,44)	1(1,64)	0	3(3,92)
Garango	165	136(82,42)	2(1,21)	5(3,03)	22(13,33)
Koupéla	256	192(75)	12(4,69)	20(7,81)	32(12,50)
Ouargaye	67	59(88,06)	0	1(1,49)	7(10,45)
Pouytenga	256	209(81,64)	6(2,34)	25(9,77)	16(6,25)
Tenkodogo	330	269(81,52)	5(1,52)	22(6,67)	34(10,30)
Zabré	446	316(70,85)	36(8,07)	64(14,35)	30(6,73)
**Total région**	1581	1238(78,30)	62(3,92)	137(8,67)	144(9,11)

## Discussion

Notre étude a porté sur 1581 cas de tuberculose, incluant les nouveaux cas et les rechutes, enregistrés entre 2021 et 2023 dans neuf centres de dépistage et de traitement (CDT) répartis dans les districts sanitaires de la région du Centre-Est du Burkina Faso. Dans notre étude, la tranche d'âge la plus touchée était celle des 25-34 ans, représentant 27,8% des cas. Ce constat rejoint les données rapportées par Djika *et al*. Au Niger (23,77%) [10] et à Boualam *et al*. au Maroc (34,37%) [11]. Les patients étaient majoritairement de sexe masculin (sex-ratio de 3,8). Cette prédominance masculine pourrait s'expliquer par une plus grande exposition aux facteurs de risque professionnels. En effet, 31,8% des cas exerçaient dans l'orpaillage, une activité à haut risque de transmission de la tuberculose en raison des conditions de vie précaires et de la promiscuité dans les sites miniers, comme l'ont montré Edwige *et al*. [12] et Moyo *et al*. [13]. L'agriculture représentait 27,6% des cas. Ce secteur expose également les travailleurs à des facteurs environnementaux défavorables, comme l'ont souligné Mia *et al*. [14] et Ngwira *et al*. [15].

Les districts de Zabré (25,2%) et Tenkodogo (19,3%) ont enregistré le plus de cas de tuberculose. Cette situation pourrait s'expliquer par une meilleure accessibilité des structures de santé, la présence de sites d'orpaillage et l'accueil de nombreuses personnes déplacées internes (PDI). À l'inverse, les districts d’Ouargaye et Bittou ont enregistré une faible notification, probablement liée à la dégradation sécuritaire, la fermeture des formations sanitaires, des sites d'orpaillages et les mouvements de population. La proportion de co-infection tuberculose-VIH observée dans notre étude était de 6,01%. Elle demeure inférieure à celles rapportées par Kalombo *et al*. en République démocratique du Congo (10,7%) [16] et par Ka *et al*. au Sénégal (10%) [17]. Cette faible proportion pourrait s'expliquer par des difficultés d'accès au dépistage du VIH dans certaines zones à fort défi sécuritaire, notamment à Bittou et Ouargaye, où les ruptures d'intrants sont fréquentes en raison des problèmes de réapprovisionnement. À cela s'ajoute une possible réticence de certains patients à se faire dépister. Parmi les patients co-infectés, la tuberculose pulmonaire représentait 88,3% des cas. Nos résultats sont similaires à ceux rapportés par Mouchrik *et al*. au Maroc, où 89% des cas étaient des formes pulmonaires confirmées microscopiquement [18]. Ils sont également comparables à ceux de Zhang *et al*. en Chine, qui ont observé une prédominance de ces formes chez les patients immunodéprimés [19]. Le taux de succès du traitement observé dans notre étude (78,3%) reste inférieur à l'objectif de 85% recommandé par l'Organisation mondiale de la Santé (OMS) [2]. Dans les districts fortement affectés par l'insécurité, tels qu'Ouargaye et Bittou, la fermeture des centres de santé, le déplacement des populations et l'insuffisance du suivi communautaire ont contribué à un taux d'abandon de 8,67% et une létalité de 9,11%. Par ailleurs, la présence de nombreux sites d'orpaillage dans la région représente un facteur aggravant. Plusieurs patients interrompent prématurément leur traitement dès l'amélioration de leur état pour aller travailler dans les mines. Cette dynamique compromet la complétude des cures, accroît le risque d'échec thérapeutique et favorise la transmission continue de la maladie. Ces résultats sont comparables à ceux rapportés par Shaweno *et al*. en Éthiopie, qui ont observé un taux de succès thérapeutique de 77,3% chez les patients co-infectés VIH/tuberculose dans un contexte de vulnérabilité accrue [20]. Des performances supérieures ont été rapportées dans des contextes plus stables, notamment au Maroc où Mouchrik *et al*. ont documenté un taux de succès de 84,13% [18], et au Cameroun, où Chethkwo *et al*. ont rapporté un taux de succès thérapeutique de 87,3% [21]. La qualité du suivi des patients, l'accessibilité aux services de santé, la disponibilité des intrants (notamment les antituberculeux et les fiches de suivi), ainsi que l'implication du personnel communautaire sont des facteurs déterminants pour atteindre les objectifs thérapeutiques. L'OMS recommande également l'utilisation d'outils numériques simples (téléphonie mobile, rappels par SMS) pour améliorer l'observance thérapeutique dans les milieux à ressources limitées [22].

L'incidence de la tuberculose est passée de 29,31 à 33,30 cas pour 100000 habitants entre 2021 et 2023, traduisant une tendance globale à la hausse. Cette augmentation pourrait traduire un renforcement des capacités de détection, notamment par la mise en œuvre en 2022 de la stratégie REATB (recherche active de la tuberculose) dans les hôpitaux de district et en milieu carcéral, la formation du personnel soignant et la dotation des CDT en équipements et intrants. Toutefois, elle pourrait aussi refléter une recrudescence réelle de la maladie dans un contexte de vulnérabilité croissante, aggravé par la précarité, la promiscuité et les déplacements de population. L'analyse des pics saisonniers (février-avril et septembre-novembre) suggère des périodes où la transmission ou le dépistage est accru. Ces variations pourraient être liées aux cycles agricoles, aux migrations saisonnières, aux conditions climatiques ou aux campagnes de dépistage organisées par les services de santé. Des études complémentaires seraient nécessaires pour mieux comprendre l'influence de ces facteurs sur la dynamique de la tuberculose dans la région.

**Limites de l'étude:** la principale limite de notre étude réside dans sa conception transversale descriptive, qui ne permet pas d'établir de relation causale entre les facteurs étudiés et la survenue de la tuberculose. De plus, le contexte sécuritaire instable a pu influencer la fréquentation des structures sanitaires, ce qui pourrait entraîner une sous-estimation du nombre réel de cas. Cependant, la couverture exhaustive de tous les CDT de la région et le contrôle de qualité effectué lors de la saisie des données renforcent la fiabilité des résultats.

## Conclusion

Cette étude a mis en évidence le fardeau de la tuberculose dans la région du Centre-Est du Burkina Faso, particulièrement chez les jeunes hommes exerçant des activités à risque comme l'orpaillage et l'agriculture. Les performances thérapeutiques observées demeurent insuffisantes et inférieures aux objectifs fixés. Dans un contexte sécuritaire difficile, le renforcement du dépistage actif, l'intégration des soins TB/VIH et le soutien communautaire apparaissent comme des leviers essentiels pour améliorer la prise en charge des patients.

### Etat des connaissances sur le sujet

- La tuberculose est une maladie endémique au Burkina Faso, affectant particulièrement les régions rurales;
- Les contextes d'insécurité compromettent l'accès aux soins et la continuité du traitement;
- Les activités comme l'orpaillage sont associées à un risque accru de tuberculose.

### Contribution de notre étude à la connaissance

- Une analyse détaillée du profil épidémiologique de la tuberculose dans une région à fort défi sécuritaire;
- Une mise en évidence des pics saisonniers de cas, suggérant une dynamique particulière de transmission;
- Des recommandations adaptées à un contexte de crise pour améliorer la détection et la prise en charge.
